# The Impact on Dietary Outcomes of Celebrities and Influencers in Marketing Unhealthy Foods to Children: A Systematic Review and Meta-Analysis

**DOI:** 10.3390/nu14030434

**Published:** 2022-01-19

**Authors:** Jessica Packer, Simon J. Russell, Gabriela Siovolgyi, Katie McLaren, Claire Stansfield, Russell M. Viner, Helen Croker

**Affiliations:** 1 Population, Policy and Practice Research and Teaching Department, UCL Great Ormond Street Institute of Child Health, University College London, London WC1N 1EH, UK; s.russell@ucl.ac.uk (S.J.R.); gabrielansiovolgyi@gmail.com (G.S.); katie.mclaren@hotmail.co.uk (K.M.); r.viner@ucl.ac.uk (R.M.V.); h.croker@wcrf.org (H.C.); 2 EPPI-Centre, UCL Social Research Institute, University College London, London WC1H 0NR, UK; c.stansfield@ucl.ac.uk

**Keywords:** child and adolescent health, food marketing, obesity, policy research

## Abstract

Celebrities, including influencers, are commonly used to market products that are high in fat, sugar, and salt (HFSS) to children but the impact on dietary outcomes has been unclear. The primary aim of this study was to systematically review the literature and quantify the impact of celebrities in HFSS marketing on children’s dietary outcomes. We searched eight databases and included studies from all countries and languages published from 2009 until August 2021. Participants were defined as under 16 years, exposure was marketing for HFSS products with a celebrity, and the outcomes were dietary preference, purchasing behaviors, and consumption of HFSS products. We were able to conduct a meta-analysis for consumption outcomes. Seven articles met the inclusion criteria, of which three were included in the meta-analysis. Under experimental conditions, the use of celebrities in HFSS marketing compared to non-food marketing was found to significantly increase consumption of the marketed HFSS product by 56.4 kcals (*p* = 0.021). There was limited evidence on the impact on preference or purchase intentions and on the comparisons between use and non-use of celebrities and influencers.

## 1. Introduction

Increased exposure to high in fat, sugar, and salt (HFSS) marketing is occurring simultaneously with the global childhood obesity epidemic [1]. The marketing of unhealthy foods is ubiquitous and is particularly impactful for children and young people (the term ‘marketing’ includes both advertising and packaging) [2]. Children are exposed to marketing throughout the food environment, including via television and other broadcast media, in shops and supermarkets, on the street, and, increasingly, online [3]. The majority of food marketing is for HFSS products, some of which is directly targeted at children [4,5]. Evidence suggests that children from ethnic minority and lower socioeconomic groups are disproportionately exposed to, and influenced by, food marketing [6]. Children with higher body weight have also been found to be disproportionately affected by screen advertising for HFSS products [7].

The need for enhanced regulations of commercial marketing is a priority on the global health and policy agenda, as highlighted in a recent WHO–UNICEF–Lancet child health Commission [8]. This follows on from the 2010 WHO recommendations, for policies to limit the effectiveness of HFSS food marketing to children by limiting its exposure and power (the creative content, design, and execution of the marketing message/impacted by techniques used) [9]. In the accompanying WHO implementation guidance, restricting the use of celebrities in HFSS product marketing was used as a specific example of how to reduce the power of marketing [10]. Celebrity endorsements, across a broad array of categories, including HFSS food products, have been shown to increase sales, bring about more positive attitudes to brand and product, and increase purchase intentions [11,12,13,14].

Celebrity endorsements are thought to work through the process of evaluative conditioning, where liking of a stimulus is the result of its pairing with other positive stimuli [15]. This process is mediated by the parasocial relationships children can form with celebrities (one-sided relationships between media users and celebrities), especially through social media interaction [16]. A cross-disciplinary review examining how celebrities influence patients’ health-related behaviors found that they can help distinguish products and elicit herd behavior (economics); transfer positive characteristics to the endorsed products (marketing); activate brain regions associated with trust, creating positive associations and encoding memories (neuroscience); and cause positive reactions (psychology) [17]. This results in the possibility of celebrities having a substantial influence on people’s health-related behaviors.

Current restrictions on the use of celebrities in marketing of HFSS products to children have been identified as an area of concern [18,19,20]. Whilst the UK, Ireland, Chile, Australia, Netherlands, Portugal, Spain, and Brazil restrict the use of celebrities in HFSS advertising to children, loopholes exist in the interpretation of defining celebrities and audience thresholds, and the implementation of regulations [19,21,22,23,24]. In the UK, for example, the use of celebrities ‘popular with children’ is restricted in broadcast and non-broadcast HFSS advertisements targeting pre- and primary school children (under 12 years) but no definition of what constitutes a celebrity ‘popular with children’ is provided [25,26]. Additionally, the regulation of restrictions varies between self-regulated or statutory legislation. Evidence has shown that self-regulation is broadly ineffective at limiting HFSS marketing to children [19,27,28,29]. An example of voluntary and self-regulated restrictions is the Spanish Publicidad, Actividad, Obesidad, Salud (PAOS) Code for food and drinks marketing to children, which prevents the participation, appearance, and exploitation of well-known and famous persons [30]. The scope of restrictions is mainly focused on broadcast marketing, with packaging, sponsorship, cinema, and in-store promotions commonly neglected [18,19]. Restrictions frequently apply only to pre-digital media and need to be updated to react to changes in marketing and media consumption, with digital marketing now accounting for the majority of UK advertising spend [18,31]. Children as young as 3–4 years old are increasingly switching their preference and usage from TV to online (e.g., YouTube), and YouTube is growing as the preferred platform [3]. This has led to a new type of celebrity, the ‘influencer’ (or YouTuber), defined as gaining fame by successfully branding themselves as experts on social media platforms [32], which has been identified as a new marketing source targeting children [33]. Experiments have shown that influencer marketing leads to greater purchase intentions due to participants identifying, relating to and trusting influencers more than other celebrities [32]. Evidence suggests that the integration of ‘real-life’ scenarios into social media marketing (use of advertised product in their daily lives) leads to greater positive brand effects (brand attitude, purchase intention, willingness to pay for a product, and feeling connected to the brand) compared to traditional commercial celebrity-endorsed advertising [34]. Sports celebrities are also of interest in our review, with research showing that their association with high-sugar products can foster beliefs in children that they are healthy and improve sports performance [35].

Content analyses show that food marketing featuring celebrities is particularly prominent for HFSS products [36,37,38,39]. This was consistent across television advertisements in the UK [36] and musician [37], athlete [38], and YouTube influencer endorsements in the US [39]. Celebrities are also used on HFSS product packaging [40]. Analysis of social media advertising exposure in children aged 7–16 years found that during 10 min of social media use, 72% were exposed to food advertising, primarily for HFSS products, of which 17% was embedded in celebrity generated content [41]. Embedded content is not explicitly advertising, adding to the difficulty children already face in recognizing online advertising and impacting their ability to understand the intent of advertising [42,43]. A longitudinal study, looking at the association between self-reported vlog (i.e., video weblogs) viewing and consumption of HFSS beverages or snacks at three time points, found a significant association between exposure and consumption of HFSS beverages 24 months later [44].

Despite the widespread use of celebrities in the marketing of HFSS products and given the evidence of their impact on dietary outcomes, there have been no systematic reviews to date examining their impact on preference, purchasing, and consumption outcomes in children. Reviews have reported that celebrities are a popular marketing tactic for promoting HFSS foods to children but impacts on outcomes have rarely been assessed [45,46]. One review examined the impact of food marketing tactics on children’s attitudes, preferences, and consumption and included the use of endorsers but found only limited evidence relating to advertising with celebrities [47]. Another review found evidence that celebrity endorsements positively impact brand attitudes and purchase intentions but included only one study in adolescents [11]. Due to these gaps in the literature, we undertook a review to better understand how celebrities used in HFSS food marketing (advertising and packaging) impact on children’s food preferences, purchasing behaviors, and consumption. Our secondary aims were to assess the differential impact of the type of celebrity (sports, YouTubers/influencers, or other), child characteristics (age and socio-economic status), format of advertisement (content within advertisements versus on packaging), and length of any effects (short- or long-term).

## 2. Materials and Methods

### 2.1. Protocol and Registration

The current systematic review and meta-analysis was conducted and reported in accordance with the PRISMA statement checklist and was registered with PROSPERO (CRD42019155037) [48].

### 2.2. Eligibility Criteria, Information Resources, and Search Strategy

To be eligible for inclusion, studies needed to be quantitative, experimental (randomized or non-randomized) with an advertising exposure featuring a celebrity and a comparison group (non-food advertisement, no exposure, healthy food advertisement), or “real-world” (longitudinal, interrupted time series, controlled before and after). Studies from 2009, in any country or language, were included, as well as both between-subject and within-subject designs. Studies before 2009 were excluded as they were likely to be of limited relevance due to rapid advancement of technology and celebrity/influencer culture. Participant criteria were children aged between 0 and 15 years, in line with UK advertising regulations. Any marketing modality (TV, online, internet/advergames, poster, packaging, digital advertising) with a celebrity/influencer was eligible. Outcomes were all related to the advertised HFSS food product and included consumption (measured dietary intake, ad libitum consumption), preferences (self-reported, like/dislike ratings), and purchasing/request (quantity of product purchased, pester intention).

Searches of the following electronic databases were conducted on 22 October 2019 and updated on 16 August 2021: Ovid MEDLINE, Cochrane Library, Scopus, PsycINFO, ProQuest (Central)—ASSIA, Web of Science—Social Science Citation Index and Emerging Sources Citation Index and Social Policy and Practice (see Supplementary Table S1 for full search details and Supplementary Table S2 for search strings). K.M., G.S., and J.P. conducted the searches, imported records into EndNoteX9 and EPPI-Reviewer 4 and removed duplicates. EPPI-Reviewer 4 was used for screening and for search management [49].

### 2.3. Study Selection

Exclusion criteria were date (published pre-2009), participant age group (over 16 years), study design (qualitative, content analyses, cross-sectional), publication type (reviews, dissertations), intervention (no HFSS marketing exposure with a celebrity), and outcome measure (no measure of food intake, consumption, choice, preference, purchase, purchase intention, or pestering).

Double screening of papers on title and abstract and full-text were independently completed by K.M., G.S., J.W., and J.P. Discrepancies between reviewers were mutually reconciled. Full-texts of relevant articles were acquired via library and web services, in addition to direct contact with authors. All papers eligible for screening were retrieved successfully.

### 2.4. Data Extraction and Items

Data were independently extracted and jointly reconciled by K.M., G.S., and J.P. Corresponding authors were contacted to request additional data, where required, for the meta-analysis. Four corresponding authors were contacted, of whom one responded with the required data.

Data extracted included study identification (authors, country, year of publication), target population (children and/or adolescents), sample group description (size of sample, age range, and mean age of participants), study description (study design, number of participants in each condition and assignment to conditions), intervention description (advertising medium, celebrity/influencer), comparison type (HFSS food advertisement vs. healthy food or non-food advertisement), test foods used, outcome type (consumption, preference, or purchasing), and outcome measures (kcals, kJ, grams, preference ratings, purchase request measures).

### 2.5. Assessment of Quality

The Cochrane risk of bias tool RoB 2 was used to assess bias in the included experimental studies [50]. Bias assessment was conducted independently by two reviewers with discrepancies reconciled.

### 2.6. Data Synthesis

Our primary analysis was meta-analysis, but where studies did not provide sufficient data, they were included in a narrative synthesis. For inclusion in the meta-analysis, experimental studies were required to have an appropriate comparison group, including no advertisement or non-food advertisement. We considered these comparison groups due to the ubiquity of using celebrities in marketing of both food and non-food products and to be consistent with previous research [20]. Due to a lack of studies measuring preference or purchase of a HFSS product, meta-analysis was only possible for consumption outcomes. Three articles were identified that measured HFSS consumption and reported/provided the mean values with standard deviations. The consumption outcomes were standardized to report the total energy content consumed (kcals), which required conversion from weight (grams) using published nutritional values of the consumed products. Further details about the standardization methods, and the rationale for the experimental conditions and outcomes, were included in the meta-analysis, is provided in a supplemental file (Supplementary Table S3). Due to heterogeneity in study design, including the type of celebrity (sport or influencer), the advertising exposure (online static image, TV, or YouTube clip), and the HFSS products advertised and consumed (crisps or chocolate biscuit), a DerSimonian-Laird random-effects model was used for meta-analysis. We presented the results graphically using forest plots. Analyses were conducted using Stata 16 (16.1, StataCorp LLC, College Station, TX, USA) [51].

## 3. Results

### 3.1. Study Selection

**Figure 1:**
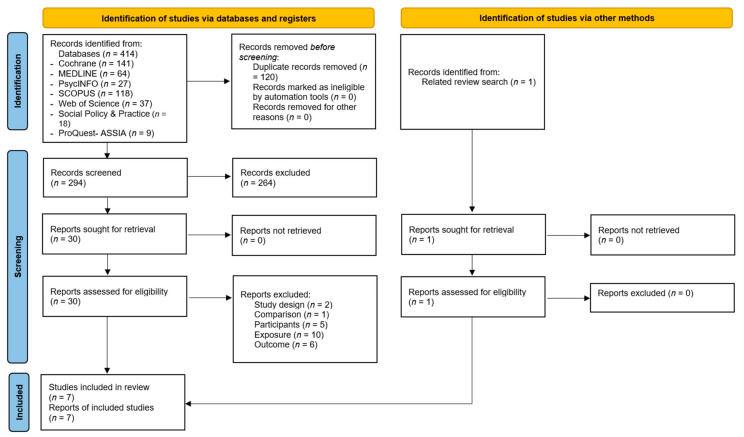
PRISMA screening flowchart.

The search process is shown in the flowchart (Figure 1). The searches resulted in 414 articles, of which 294 were unique records following the removal of duplicates. After screening on title and abstract, 264 were excluded and 30 were screened on full-text. One article was found through screening of a related review. This led to the inclusion of seven studies, from seven reports. Three studies were included in the meta-analysis.

### 3.2. Study Description and Results

A summary of study information is provided in Table 1.

### 3.3. Participants

The age of participants across all studies ranged from 6–17 years. One study included participants aged 13–17, and results were not split by age [56]. The range for the three studies in the meta-analysis was 8 to 11 years, and the mean age was 10.3 years.

### 3.4. Settings

All studies were experimental using between-subjects designs. All but two [20,56] stated that subjects were randomly allocated. All of the studies were conducted in schools or education centers. Studies were conducted in the UK (*n* = 3, same research group), India (*n* = 1), Belgium (*n* = 1), Australia (*n* = 1), and Spain (*n* = 1).

### 3.5. Interventions

The HFSS marketing exposure varied: TV advertisements, embedded in cartoons (*n* = 2) [20,57], an online advertisement embedded in a YouTube clip (*n* = 1) [52]; static Instagram posts (*n* = 2) [53,54]; food product packaging (*n* = 1) [55]; and printed advertisements (*n* = 1) [56]. The celebrities featured influencers (*n* = 3), sports celebrities (*n* = 3), and a Hindi movie star (*n* = 1). The HFSS products included crisps, chocolate, chocolate biscuits, donuts, and sweetened cereals.

### 3.6. Outcomes

The outcome measures were ad libitum consumption of snacks immediately following the advertising exposure (from which total calories consumed could be calculated) and self-reported consumption intention (*n* = 3) [20,52,53], preference/snack choice between paired items (*n* = 3) [54,55,57], and self-reported purchase intention (*n* = 1) [56]. The HFSS products available for ad libitum consumption were crisps, chocolate biscuits, and jelly candy/chocolate buttons. Duration of snacking, when reported, was between 5–10 min.

### 3.7. Comparisons

Calorie consumption following viewing of advertisements of HFSS products with celebrities was compared to consumption following non-food advertisements with or without celebrity endorsements in the three studies included in the meta-analysis [20,52,53]. Of the studies not included in the meta-analysis, two studies compared marketing of a HFSS product with celebrity endorsement to the same HFSS product without celebrity endorsement packaging [55] or print advertisement [56]; one study compared TV advertisements with celebrity endorsement for HFSS product to non-exposure or non-food TV advertisements [57]; and one study compared Instagram posts with influencers for HFSS products to Instagram posts with influencers for non-HFSS food products [54].

### 3.8. Meta-Analysis

**Figure 2:**
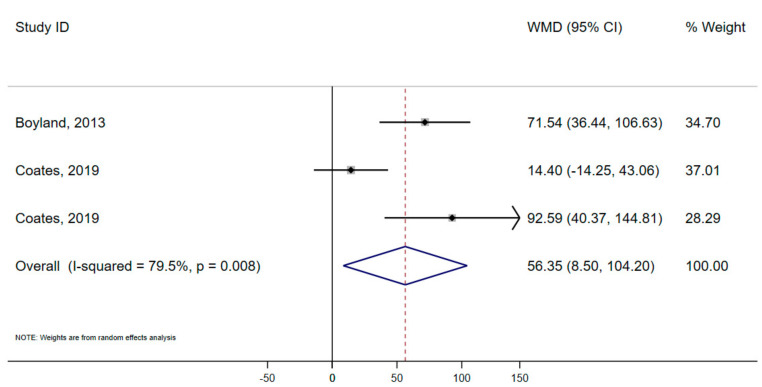
Forest plot showing mean difference (kcals) in total snack consumption of HFSS products between celebrity HFSS advertisement and non-food advertisement. Boyland, 2013; Coates, 2019; Coates, 2019.

Three studies provided sufficient data on calorie consumption to be included in meta-analysis of consumption. We found that use of a celebrity in HFSS food marketing, compared to non-food marketing, resulted in significantly greater consumption of HFSS products, with a pooled effect size of 56.4 kcals (95% CI 8.50, 104.20; *p* = 0.021) (Figure 2). We found evidence of high heterogeneity (I^2^ = 79.5%); Egger’s regression analysis showed low risk of publication bias (*p* = 0.347); and trim and fill analysis suggested evidence of two missing studies (See Supplementary Figure S1).

### 3.9. Other Findings

One study measured purchase intentions and found purchase intentions for HFSS products were significantly greater in the celebrity HFSS advertisement group compared to non-food advertisement [56]. Preference was measured in three studies, with mixed results [55]. One found that when exposed to product packaging featuring an endorsement from a sports celebrity that product was chosen by participants significantly more when compared to the same product with no endorsement; however, this effect was only seen in boys [55]. Two studies found that preference for HFSS product was not significantly different with celebrity-endorsed HFSS product advertisement exposure compared to no advertisement exposure or non-food advertisement control [57] or non-HFSS food product [54]. The impact of SES was only reported in one study, which found no association between SES and food preference in response to HFSS advertising [55]. Impact of age was measured in three studies and was found to not be significantly associated with consumption (*n* = 2) [20,52] or consumption preference [57]. We were unable to assess the secondary aims of differential impact of celebrity type or format of advertisement, as the data did not support this. None of the included studies were longitudinal; therefore, long-term consequences of these effects could also not be assessed. Across all studies, there was little evidence relating to comparisons between use and non-use of celebrities and influencers (i.e., endorsed vs. non-endorsed) in HFSS food marketing.

### 3.10. Quality of Studies

The risk of bias across included studies was assessed as mostly low (see Supplementary Figure S2 for bias assessment).

## 4. Discussion

Our systematic review included the first meta-analysis examining the impact of celebrities used in the marketing of HFSS products to children and found evidence that marketing HFSS products with celebrities’ influences children’s calorie consumption. The meta-analysis showed that HFSS advertisements with a celebrity endorser, compared to a non-food advertisement, resulted in significantly greater calorie intake in children under experimental conditions. We found limited evidence that celebrities impact purchase intentions and mixed evidence that they impact preference outcomes. The findings from our review extend the findings of previous reviews, which have indicated that celebrities are persuasive HFSS marketing tools [11,45,46,47]. Our previous work showed that, following exposure to screen advertising for food, children consumed an additional 57 kcals [7] when compared to non-food advertisement exposure, which is consistent with the finding here of an additional 56.4 kcals. The impacts of advertising may be modest but over time can accrue to have substantial impacts on energy balance, body weight, and associated morbidities [58,59].

There was limited evidence that age did not impact on consumption outcomes but only one study examined the impact of SES and found no evidence. A review recently found that children from ethnic minority and low SES backgrounds are exposed to more HFSS advertising than children from higher SES and non-ethnic minority backgrounds [6]. This emphasizes the need for policy actions that addresses these inequalities.

Our data suggest there is potential for population-level interventions, including policy action such as enhanced regulations, to have an impact on HFSS consumption by children, even if effects are modest at an individual level. The use of celebrities in HFSS marketing is often not restricted or subject to weak regulations, and greater policy action has been recommended by WHO. The quantifiable impact of celebrities on children’s dietary outcomes has not been previously evidenced in the literature [18,19]. Our findings suggest that tightening policies regulating HFSS marketing directed at children that contain celebrities may be effective in reducing children’s calorie intake. Celebrity-endorsed HFSS brand advertising is frequently omitted from regulations, due to complexities in identifying advertised products and assessing if restrictions are applicable [60]. An additional concern with using celebrities in HFSS marketing is the knock-on effect of exposure to them in contexts outside of HFSS advertisements. This has been shown to increase consumption of the HFSS food (seeing Gary Lineker in Match of the Day led to children eating more Walkers crisps) [20]. Chile has implemented policies that comprehensively restrict use of celebrities, and these have been shown to be effective at reducing HFSS food marketing to children [19,60,61]. The UK government announced plans to introduce a pre-watershed ban on HFSS advertisements across television and on-demand program services, and a restriction on paid-for less healthy food and drink advertising online, which could be effective at overcoming some gaps and limiting exposure of celebrities in HFSS marketing to children [60,61]. These policies touch on areas where regulations could be strengthened including a standardization of approaches to achieve a consistent definition of celebrities, scope (programs and medias), audience thresholds (approved composition of the audience i.e., proportion of children), and enforcement. In the UK, only celebrities ‘popular with children’ are restricted (but this is not defined), allowing free use of celebrities with general appeal, such as Gary Lineker, and unrestricted endorsement opportunities for influencers [18,19]. Certain regulations (e.g., Ireland, UK, and the EU pledge) only apply if children make up between 20–50% of the viewing audience, but quantifying the audience demographics for broadcast and non-broadcast media is difficult to accurately assess [18]. This is especially true online, where user age restrictions on social media platforms are rarely followed by site users and children often use their parents’ account or devices [3]. Restrictions specific to children’s programs, mean that family programs popular with children and broadcast during peak children viewing times (6–9 p.m. in the UK) are out of scope in many jurisdictions but could be overcome by total bans on HFSS advertising, such as the proposed pre-watershed ban in the UK [3,29].

Limitations of this review include the small number of search results and limited number of studies eligible for inclusion in meta-analysis; therefore, care needs to be taken due to variability. The heterogeneity of the included studies was high, but the random effects model was used to account for differences. Findings from the meta-analysis should be interpreted with caution, as all studies were completed by the same research team at the University of Liverpool but conducted to a high standard. Further primary research would be beneficial in building the evidence base, especially digital marketing. We were unable to address the secondary aims, due to a lack of data. Despite our search including real-world studies, we were unable to identify any and therefore were unable to investigate the long-term impacts of celebrity endorsers on dietary outcomes. Beyond age, we were unable to assess the influence of SES on impact of advertising and in future would also examine the impact of weight status and ethnicity. We were unable to assess if there were any changes due to COVID-19, as no papers specifically mention this and most of the data was collected pre-2020. Due to the data available, we were unable to complete meta-analysis comparing use and non-use of celebrities and influencers in HFSS marketing and the impact on dietary outcomes (i.e., endorsed vs. non-endorsed). We suggest this as an area for future primary research; it is also a limitation about the difficulty of separating the effect of celebrity from marketing more generally. The impact of celebrity and influencers on promoting healthier food products was not the focus of this review and could be another area of future research. Evidence suggests food adverts do not appear to work in the same way for healthy food [62].

## 5. Conclusions

We found evidence that HFSS food marketing featuring celebrities or influencers increases children’s food consumption, although this was from a limited number of studies. These findings suggest that limiting exposure of children to HFSS marketing including all celebrity types may have beneficial impacts upon dietary consumption. Further research on the impact of child characteristics such as SES, long-term impacts, and real-world studies would be beneficial to further inform the thinking of policy makers.

## Figures and Tables

**Table 1:** Descriptive table of experimental studies.

Author, Year, Country	Participants	Design	Advertising Intervention	Comparison	Outcome	Relevant Results	Risk of Bias
Boyland [20], 2013, UK	*N* = 181 Age range = 8–11 Mean age = 10.3	Experimental (school), between-subject, allocation not specified	20 min cartoon with 45 s TV advert for HFSS product (Walker’s crisps) with sports celebrity endorser (Gary Lineker)	20 min cartoon with 45 s non-food advert; food advert with no endorser; or TV footage of endorser	Post-intervention, ad libitum consumption of potato crisps, labeled branded, and non-branded (grams)	Celebrity endorsed TV food adverts significantly increased intake of food, compared to food advert with no endorser and non-food advert.	Some concerns
Coates [52], 2019, UK EoI	*N* = 151 Age range = 9–11 Mean age = 10	Experimental (school), between-subject, random assignment	5 min YouTube video with 1 min influencer marketing (Zoella and PointlessBlog) segment of branded HFSS product (McVitie’s chocolate biscuits), with and without disclosure	5 min YouTube video with 1 min influencer marketing segment of branded non-food product (Apple iPhone)	Post-intervention, ad libitum consumption of cookies (kcal), labeled, branded, and non-branded, 5 min	Influencer endorsed HFSS advert significantly increased intake of promoted food, compared to non-food advert	Low
Coates [53], 2019, UK SMI	*N* = 176 Age range = 9–11 Mean age = 10.5	Experimental (school), between-subject, random assignment	1 min viewing of mock Instagram profile of popular YouTube influencers (not stated due to copyright) with marketing of HFSS product (unbranded chocolate biscuits)	1 min viewing of mock Instagram with image of YouTube influencer marketing healthy product (banana) or non-food (sneakers)	Post-intervention, ad libitum consumption of unbranded HFSS products HFSS (candy, chocolate) and healthy (carrot, grapes) products (kcal), 10 min	Intake of HFSS products and overall snacks significantly increased following exposure to celebrity endorsement of HFSS products, compared to non-food condition.	Low
De Jans [54], 2021, Belgium	*N* = 190 Age range = 8–12 Mean age = 10.04	Experimental (classroom), between-subject, random	Instagram post of influencer (fictitious) promotion of HFSS snack (unbranded donuts) (either sedentary lifestyle versus athletic lifestyle)	Instagram post of influencer promotion of snack high in nutritional value (strawberries) (both (influencer lifestyle: sedentary versus athletic)	Snack choice between mini donut or a strawberry.	Children exposed to influencer promotion of the donut, chose the donut 52.2% (47/90) compared to 49.5% exposed to influencer promotion of non-HFSS product. Significance not tested.	Low
Dixon [55], 2014, Australia	*N* = 1302 Age range = 10–12 Mean age = 11	Experimental (online school), between-subject, random assignment	Packaging of HFSS products (cereal, cheese dips, chicken nuggets, ice cream, flavored milk, brands not stated) with sports celebrity endorsement (popular Australian male athletes, names not stated)	Packaging of same HFSS products with no celebrity endorsement (no promotion)	During-intervention, forced choice of randomly allocated HFSS exposure or comparable healthier food pack, on a computer	Celebrity endorsed HFSS products were significantly more likely to be chosen compared to control, in boys only. No significant difference in girls.	Low
Jain [56], 2011, India	*N* = 378 Age range = 13–17 Mean age = not stated	Experimental (school), between-subject, allocation not specified	5–10 min viewing of print advertisement of HFSS product (unbranded chocolate) with celebrity endorsement (Hindi actor, Aamir Khan)	5–10 min viewing of print adverts of HFSS product with no endorsement	Post-intervention, purchase intention product (scale NS)	Purchase intentions of HFSS product endorsed by a celebrity were significantly greater compared to control or character-endorsed HFSS product.	Some concerns
Ponce-Blandon [57], 2020, Spain	*N* = 421 Age range = 4–6 Mean age = 4.8	Experimental (education centers), between-subject, random assignment	8 min episode of cartoon (Caillou) with an advert for HFSS product (Príncipe Double Choc chocolate cookies) with sports celebrity (famous Spanish soccer player, name not stated)	No advert control and non-food advert control	Preference choice between advertised product (Príncipe Double Choc chocolate cookies) vs. similar non advertised product (Tosta Rica Chocoguay, Cuétara chocolate cream filled cookies)	Preference for the advertised product was not significantly different between the conditions.	Low

## Data Availability

The data presented in this study are available in Boyland [20], 2013; Coates [52], 2019; Coates [53], 2019.

## Supplementary Materials

The following supporting information can be downloaded at: https://www.mdpi.com/article/10.3390/nu14030434/s1, Table S1: Details of search, Table S2: Search history, Table S3: Rationale for meta-analysis inclusion and data processing, Figure S1: Trim and fill analysis, Figure S2: Bias assessment for experimental studies
