# Supplementary material for: The Impact on Dietary Outcomes of Celebrities and Influencers in Marketing Unhealthy Foods to Children: A Systematic Review and Meta-Analysis
Source: Nutrients. 2022 Jan 19;14(3):434. doi: 10.3390/nu14030434 (PMC8837952; doi:10.3390/nu14030434)

Supplemental file

Table S1: Details of search

Databases

- Medline (OVID)
- Cochrane Library
- Scopus
- PsycInfo (OVID)
- ProQuest (Central)- ASSIA
- Web of Science- Social sciences and emerging sources citation indexes
- Social Policy and Practice (OVID)

Inclusion criteria/search terms

**Participants**	**0-15 years**	**child* OR youth* OR adolescen* OR young people OR young person* OR schoolchild* OR boy* OR girl* OR teen* OR school child* NOT adult***
**Exposure (intervention)**	Exposure to advertising of HFSS/unhealthy food advertisement	advert* OR market* OR commercial* OR promot* OR Instagram* OR youtube* OR facebook* OR pack * OR label*, OR sticker* OR bag* OR box* OR tin* OR jar* OR carton* OR sleeve*, OR advergam* OR tag* OR lid* OR container*
**Exposure (celebrities/influencers)**	Exposure to advertising of HFSS/unhealthy food advertisement using celebrities/influencers	celebrit* OR celebrity endors* OR endors* OR influenc* OR social media influenc* OR social influenc*
**Exposure (food)**	Intervention vs. no intervention<br>Comparison of HFSS food advertisement with celebrities/influencer and non-food advertisement control OR<br>HFSS food advertisement with celebrity/influencer compared to HFSS food advertisement without celebrity/influencer<br>Could also include a healthy food comparison group with OR without celebrity/influencer.	food* OR beverage* OR soda* OR cola* OR fizzy adj drink* OR carbonated adj beverage* OR carbonated adj drink* OR soft adj beverage* OR soft adj drink* OR high salt* OR high fat* OR high sugar* OR snack* OR sweet* OR meal* OR HFSS OR snack* OR sweet* NOT alcohol*
**Outcome measure**	Any of these:<br>Energy intake<br>Ad libitum consumption of food<br>Dietary behaviour<br>Preference of food<br>Purchase intention	intake* OR consumption* OR choice OR eating behavio* OR dietary intake OR body mass index OR BMI OR body composition OR adipos* OR body fat OR obes* OR overweight OR energy consumption OR energy intake OR judgement* OR preference* OR liking OR favor* OR attitude
**Study designs**	Experimental; intervention (with or without a control group)	
**Other**		

Geography	All
Languages	All
Time	2009-2021

Table S2: Search history

Medline (OVID)
1 exp adolescent/ or exp child/ or exp infant/
2 (child* or youth* or adolescen* young people or Young person* or boy* or girl* or teen* or Infant* or preschool* or toddler* or minor* or Babies or Baby).ti,ab,id.
3 1 or 2
4 Marketing/ or Advertising as topic/ or Product packaging/ or Food packaging/ or Food labelling/ or Advertisement/ or Social marketing/ or social media/
5 ((advert* adj2 food) or (advert* adj2 supermarket) or (advert* adj2 buy*) or (market* adj2 food) or (market* adj2 supermarket) or (market* adj2 buy*) or (promot* adj2 food) or (promot* adj2 supermarket) or (promot* adj2 buy*) or (promot* adj2 food) or (promot* adj2 supermarket) or (promot* adj2 buy*) or commercials or "TV commercial*" or "Television commercial*" or "radio commercial*" or "media commercial*" or digital adver* or digital market* or Mobile app* or Mobile application* or advergam* or Facebook or Instagram or Twitter or Snapchat or Youtube or Weibo or Wechat or Youku).ti,ab,id.
6 4 or 5
7 Food/ or Beverages/ or Carbonated beverages/
8 (food* or beverage* or Cola or Colas or Soda or sodas or (fizzy adj2 drink*) or (carbonated adj2 drink*) or (soft adj drink*) or high salt* or high fat* or high sugar* or snack* or sweet* or Meal* or HFSS).ti,ab,id.
9 7 or 8
10 exp Famous Persons/ or exp Athletes/
11 (celebrit* or endors* or influencer* or ((professional or elit* or famous or public or renown* or well-known or acclaim* or eminent or prominent or illustrious or recogniz* or reput* or influential or wealth* or power*) adj1 (person* or people or figure* or leader or athlete* or player or bodybuilder or sport* or basketball or football or hockey or baseball or soccer or Olympian or singer* or songwriter* or musician* or band or group or rapper* or artist* or actor* or actress or star or Hollywood or Bollywood or Nollywood or dancer or writer or author or comedian or performer or model* or supermodel* or chef or philanthropist or politic* or president or minister or king or queen or prince* or monarch))).ti,ab,id.
12 10 or 11
13 Eating/ or Snacks/ or Drinking/ or Food Preferences/ or Feeding Behavior/ or Diet, High-Fat/ or Diet/ or Diet, Western/ or energy intake/
14 (intake* or calori* or consumption* or food choice* or eating behavio* or snacking behavio* or preference* or favour* or favor* or purchas* or buy* or spend* or pester* or request* or liking).ti,ab,id.
15 13 or 14
16 3 and 6 and 9 and 12 and 15
17 16
18 limit 17 to yr="2009 -Current"

Social Policy and Practice
1 (child* or youth* or adolescen* young people or Young person* or boy* or girl* or teen* or Infant* or preschool* or toddler* or minor* or Babies or Baby).ab,de,hw,nt,ti.
2 (advert* or (market* adj2 food) or (market* adj2 supermarket) or (market* adj2 buy*) or (promot* adj2 food) or (promot* adj2 supermarket) or (promot* adj2 buy*) or (promot* adj2 food) or (promot* adj2 supermarket) or (promot* adj2 buy*) or commercials or "TV commercial*" or "Television commercial*" or "radio commercial*" or "media commercial*" or digital adver* or digital market* or Mobile app* or Mobile application* or advergam* or Facebook or Instagram or Twitter or Snapchat or Youtube or Weibo or Wechat or Youku).ab,de,hw,nt,ti.
3 (food* or beverage* or Cola or Colas or Soda or sodas or (fizzy adj2 drink*) or (carbonated adj2 drink*) or (soft adj drink*) or high salt* or high fat* or high sugar* or snack* or sweet* or Meal* or HFSS).ab,de,hw,nt,ti.
4 (celebrit* or endors* or influencer* or ((professional or elit* or famous or public or renown* or well-known or acclaim* or eminent or prominent or illustrious or recogniz* or reput* or influential or wealth* or power*) adj1 (person* or people or figure* or leader or athlete* or player or bodybuilder or sport* or basketball or football or hockey or baseball or soccer or Olympian or singer* or songwriter* or musician* or band or group or rapper* or artist* or actor* or actress or star or Hollywood or Bollywood or Nollywood or dancer or writer or author or comedian or performer or model* or supermodel* or chef or philanthropist or politic* or president or minister or king or queen or prince* or monarch))).ab,de,hw,nt,ti.

5 (intake* or calori* or consumption* or food choice* or eating behavio* or snacking behavio* or preference* or favour* or favor* or purchas* or buy* or spend* or pester* or request* or liking).ab,de,hw,nt,ti.
6 1 and 2 and 3 and 4 and 5

Web of Science
#1 TS= (child* OR youth* OR adolescen* young people OR Young person* OR boy* OR girl* OR teen* OR Infant* OR preschool* OR toddler* OR minor* OR Babies OR Baby) Indexes=SSCI, ESCI
#2 TS=(advert* or (market* adj2 food) or (market* adj2 supermarket) or (market* adj2 buy*) or (promot* adj2 food) or (promot* adj2 supermarket) or (promot* adj2 buy*) or (promot* adj2 food) or (promot* adj2 supermarket) or (promot* adj2 buy*) or commercials or "TV commercial*" or "Television commercial*" or "radio commercial*" or "media commercial*" or digital adver* or digital market* or Mobile app* or Mobile application* or advergam* or Facebook or Instagram or Twitter or Snapchat or Youtube or Weibo or Wechat or Youku)
Indexes=SSCI, ESCI
#3 TS= (food* or beverage* or Cola or Colas or Soda or sodas or (fizzy adj2 drink*) or (carbonated adj2 drink*) or (soft adj drink*) or high salt* or high fat* or high sugar* or snack* or sweet* or Meal* or HFSS) Indexes=SSCI, ESCI
#4 TS=(celebrit* or endors* or influencer* or ((professional or elit* or famous or public or renown* or well-known or acclaim* or eminent or prominent or illustrious or recogniz* or reput* or influential or wealth* or power*) adj1 (person* or people or figure* or leader or athlete* or player or bodybuilder or sport* or basketball or football or hockey or baseball or soccer or Olympian or singer* or songwriter* or musician* or band or group or rapper* or artist* or actor* or actress or star or Hollywood or Bollywood or Nollywood or dancer or writer or author or comedian or performer or model* or supermodel* or chef or philanthropist or politic* or president or minister or king or queen or prince* or monarch) Indexes=SSCI, ESCI
#5 TS= (intake* or calori* or consumption* or food choice* or eating behavio* or snacking behavio* or preference* or favour* or favor* or purchas* or buy* or spend* or pester* or request* or liking) Indexes=SSCI, ESCI
#6 ((((#5) AND #4) AND #3) AND #2) AND #1 Indexes=SSCI, ESCI

SCOPUS
1 TITLE-ABS-KEY (child* OR youth* OR adolescen* OR "young people" OR "young person*" OR boy* OR girl* OR teen* OR infant* OR preschool* OR toddler* OR minor* OR babies OR baby)
2 TITLE-ABS-KEY (advert* OR (market* W/2 food) OR (market* W/2 supermarket) OR (market* W/2 buy*) OR (promot* W/2 food) OR (promot* W/2 supermarket) OR (promot* W/2 buy*) OR commercials OR "TV commercial*" OR "Television commercial*" OR "radio commercial*" OR "media commercial*" OR (digital W/2 adver*) OR (digital W/2 market*) OR (mobile W/2 app*) OR (mobile W/2 application*) OR advergam OR facebook OR instagram OR twitter OR snapchat OR youtube OR weibo OR wechat OR youku)
3 TITLE-ABS-KEY (food* OR beverage* OR cola OR colas OR soda OR sodas OR (fizzy W/2 drink*) OR (carbonated W/2 drink*) OR (soft W/2 drink*) OR "high salt*" OR "high fat*" OR "high sugar*" OR snack* OR sweet* OR meal* OR hfss)
4 TITLE-ABS-KEY (celebrit* or endors* or influencer* or ((professional or elit* or famous or public or renown* or well-known or acclaim* or eminent or prominent or illustrious or recogniz* or reput* or influential or wealth* or power*) W/1 (person* or people or figure* or leader or athlete* or player or bodybuilder or sport* or basketball or football or hockey or baseball or soccer or Olympian or singer* or songwriter* or musician* or band or group or rapper* or artist* or actor* or actress or star or Hollywood or Bollywood or Nollywood or dancer or writer or author or comedian or performer or model* or supermodel* or chef or philanthropist or politic* or president or minister or king or queen or prince* or monarch))).
5 TITLE-ABS-KEY (intake* OR calori* OR consumption* OR "food choice*" OR "eating behavio*" OR "snacking behavio*" OR preference* OR liking OR favor* OR favour* OR purchas* OR buy* OR spend* OR request* OR pester*)
6 (TITLE-ABS-KEY ((child* OR youth* OR adolescen* OR "young people" OR "young person*" OR boy* OR girl* OR teen* OR infant* OR preschool* OR toddler* OR minor* OR babies OR baby))) AND (TITLE-ABS-KEY ((advert* OR (market* W/2 food) OR (market* W/2 supermarket) OR (market* W/2 buy*) OR (promot* W/2 food) OR (promot* W/2 supermarket) OR (promot* W/2 buy*) OR commercials OR "TV commercial*" OR "Television commercial*" OR "radio commercial*" OR "media commercial*" OR (digital W/2 adver*) OR (digital W/2 market*) OR (mobile W/2 app*) OR (mobile W/2 application* OR advergam OR facebook OR instagram OR twitter OR snapchat OR youtube OR weibo OR wechat OR youku))) AND (TITLE-ABS-KEY ((food* OR beverage* OR cola OR colas OR soda OR sodas OR (fizzy W/2 drink*) OR (carbonated W/2 drink*) OR (soft W/2 drink*) OR "high salt*" OR "high fat*" OR "high sugar*" OR snack* OR sweet* OR meal* OR hfss))) AND (TITLE-ABS-KEY ((intake* OR calori* OR consumption* OR "food choice*" OR "eating behavio*" OR "snacking behavio*" OR preference* OR liking OR favor* OR favour* OR purchas* OR buy* OR spend* OR request* OR pester*))) AND (TITLE-ABS-KEY ((celebrit* or endors* or influencer* or ((professional or elit* or

famous or public or renown* or well-known or acclaim* or eminent or prominent or illustrious or recogniz* or reput* or influential or wealth* or power*) W/1 (person* or people or figure* or leader or athlete* or player or bodybuilder or sport* or basketball or football or hockey or baseball or soccer or Olympian or singer* or songwriter* or musician* or band or group or rapper* or artist* or actor* or actress or star or Hollywood or Bollywood or Nollywood or dancer or writer or author or comedian or performer or model* or supermodel* or chef or philanthropist or politic* or president or minister or king or queen or prince* or monarch)))

PsycInfo

1 ("100" or "120" or "140" or "160" or "180" or "200").ag.

2 (child* OR youth* OR adolescen* young people OR Young person* OR boy* OR girl* OR teen* OR Infant* OR preschool* OR toddler* OR minor* OR Babies OR Baby).ti,ab,id.

3 1 or 2

4 exp Marketing/ or exp Advertising/ or exp digital marketing/ or exp social marketing/ or exp television advertising/ or commercials/ or exp media exposure/ or exp social media/

5 ((advert* or (market* adj2 food) or (market* adj2 supermarket) or (market* adj2 buy*) or (promot* adj2 food) or (promot* adj2 supermarket) or (promot* adj2 buy*) or commercials or "TV commercial*" or "Television commercial*" or "radio commercial*" or "media commercial*" or digital adver* or digital market* or mobile app* or mobile application* or advergam* or facebook or instagram or twitter or snapchat or youtube or weibo or yechat or youku).ti,ab,id.

6 4 or 5

7 Food/ or Fast food/ or exp "Beverages (Nonalcoholic)"/

8 (food* or beverage* or cola or colas or soda or sodas or (fizzy adj2 drink*) or (carbonated adj2 drink*) or (soft adj drink*) or high salt* or high fat* or high sugar* or snack or sweet* or meal or HFSS).ti,ab,id.

9 7 or 8

10 exp food intake/ or exp food preferences/ or exp "Rumination (Eating)"/ or exp eating behavior/ or exp consumer behavior/ or exp brand preferences/

11 (intake* or calori* or consumption* or food choice* or eating behavio* or snacking behavio* or preference* or "food preferences" or favour* or favor* or purchas* or "food purchas*" or buy* or spend* or pester* or request* or liking).ti,ab,id.

12 10 or 11

13 exp Famous Persons/ or exp Athletes/

14 (celebrit* or endors* or influencer* or ((professional or elit* or famous or public or renown* or well-known or acclaim* or eminent or prominent or illustrious or recogniz* or reput* or influential or wealth* or power*) adj1 (person* or people or figure* or leader or athlete* or player or bodybuilder or sport* or basketball or football or hockey or baseball or soccer or Olympian or singer* or songwriter* or musician* or band or group or rapper* or artist* or actor* or actress or star or Hollywood or Bollywood or Nollywood or dancer or writer or author or comedian or performer or model* or supermodel* or chef or philanthropist or politic* or president or minister or king or queen or prince* or monarch))).ti,ab,id.

15 13 or 14

16 3 and 6 and 9 and 12 and 15

17 limit 16 to yr="2019 -Current"

Cochrane

ID Search

#1 MeSH descriptor: [Child] explode all trees

#2 MeSH descriptor: [Infant] explode all trees

#3 MeSH descriptor: [Adolescent] explode all trees

#4 (child* or youth* or adolescen* or "young people" or "young person*" or boy* or girl* or teen* or infant* or preschool* or toddler* or minor* or babies or baby).ti,ab,id.

#5 MeSH descriptor: [Marketing] explode all trees

#6 MeSH descriptor: [Advertising as Topic] explode all trees

#7 MeSH descriptor: [Product Packaging] explode all trees

#8 MeSH descriptor: [Food Packaging] explode all trees

#9 MeSH descriptor: [Food Labeling] explode all trees

#10 MeSH descriptor: [Advertisement] explode all trees

#11 MeSH descriptor: [Social Marketing] explode all trees

#12 (advert* or (market* adj2 food) or (market* adj2 supermarket) or (market* adj2 buy*) or (promot* adj2 food) or (promot* adj2 supermarket) or (promot* adj2 buy*) or commercials or "TV commercial*" or "Television commercial*" or "radio commercial*" or "media commercial*" or digital adver* or digital market* or mobile app* or mobile application* or advergam* or facebook or instagram or twitter or snapchat or youtube or weibo or yechat or youku).ti,ab,id.
#13 MeSH descriptor: [Food] explode all trees
#14 MeSH descriptor: [Beverages] explode all trees
#15 MeSH descriptor: [Carbonated Beverages] explode all trees
#16 (food* or beverage* or cola or colas or soda or sodas or (fizzy adj2 drink*) or (carbonated adj2 drink*) or (soft adj drink*) or high salt* or high fat* or high sugar* or snack or sweet* or meal or HFSS).ti,ab,id.
#17 MeSH descriptor: [Famous Persons] explode all trees
#18 (((celebrit* or endors* or influencer* or ((professional or elit* or famous or public or renown* or well-known or acclaim* or eminent or prominent or illustrious or recogniz* or reput* or influential or wealth* or power*) adj1 (person* or people or figure* or leader or athlete* or player or bodybuilder or sport* or basketball or football or hockey or baseball or soccer or Olympian or singer* or songwriter* or musician* or band or group or rapper* or artist* or actor* or actress or star or Hollywood or Bollywood or Nollywood or dancer or writer or author or comedian or performer or model* or supermodel* or chef or philanthropist or politic* or president or minister or king or queen or prince* or monarch))))).ti,ab,id.
#19 MeSH descriptor: [Eating] explode all trees
#20 MeSH descriptor: [Feeding Behavior] explode all trees
#21 MeSH descriptor: [Snacks] explode all trees
#22 MeSH descriptor: [Drinking] explode all trees
#23 MeSH descriptor: [Food Preferences] explode all trees
#24 MeSH descriptor: [Diet] explode all trees
#25 MeSH descriptor: [Diet, High-Fat] explode all trees
#26 MeSH descriptor: [Diet, Western] explode all trees
#27 MeSH descriptor: [Energy Intake] explode all trees
#28 (intake* or calori* or consumption* or food choice* or eating behavio* or snacking behavio* or preference* or favour* or favor* or purchas* or buy* or spend* or pester* or request* or liking).ti,ab,id
#29 #1 OR #2 OR #3 OR #4
#30 #5 OR #6 OR #7 OR #8 OR #9 OR #10 OR #11 OR #12
#31 #13 OR #14 OR #15 OR #16
#32 #17 OR #18
#33 #19 OR #20 OR #21 OR #22 OR #23 OR #24 OR #25 OR #26 OR #27 OR #28
#34 #29 AND #30 AND #31 AND #32 AND #33 with Cochrane Library publication date Between Jan 2009 and Dec 2019, in Cochrane Reviews, Cochrane Protocols, Trials, Clinical Answers, Editorials, Special collections

Proquest ASSIA

1 MAINSUBJECT.EXACT.EXPLODE("child") OR MAINSUBJECT.EXACT.EXPLODE("adolescent") OR MAINSUBJECT.EXACT.EXPLODE("infant")
2 TI,AB(child* OR youth* OR adolescen* young people OR Young person* OR boy* OR girl* OR teen* OR Infant* OR preschool* OR toddler* OR minor* OR Babies OR Baby)
3 1 OR 2
4 MAINSUBJECT.EXACT.EXPLODE("Advertising") OR MAINSUBJECT.EXACT.EXPLODE("Advertisements") OR MAINSUBJECT.EXACT.EXPLODE("Marketing") OR MAINSUBJECT.EXACT.EXPLODE("Packaging")
5 TI,AB(advert* or (market* adj2 food) or (market* adj2 supermarket) or (market* adj2 buy*) or (promot* adj2 food) or (promot* adj2 supermarket) or (promot* adj2 buy*) or (promot* adj2 food) or (promot* adj2 supermarket) or (promot* adj2 buy*) or commercials or "TV commercial*" or "Television commercial*" or "radio commercial*" or "media commercial*" or digital adver* or digital market* or Mobile app* or Mobile application* or advergam* or Facebook or Instagram or Twitter or Snapchat or Youtube or Weibo or Wechat or Youku)
6 4 OR 5
7 MAINSUBJECT.EXACT("Drinks") OR MAINSUBJECT.EXACT.EXPLODE("Food")
8 TI,AB(food* or beverage* or Cola or Colas or Soda or sodas or (fizzy adj2 drink*) or (carbonated adj2 drink*) or (soft adj drink*) or high salt* or high fat* or high sugar* or snack* or sweet* or Meal* or HFSS)
9 7 OR 8

10 MAINSUBJECT.EXACT("Eating behaviour") OR MAINSUBJECT.EXACT "Food consumption")
11 TI,AB(intake* or calori* or consumption* or food choice* or eating behavio* or snacking behavio* or preference* or favour* or favor* or purchas* or buy* or spend* or pester* or request* or liking)
12 10 OR 11
13 TI,AB(celebrit* or endors* or influencer* or ((professional or elit* or famous or public or renown* or well-known or acclaim* or eminent or prominent or illustrious or recogniz* or reput* or influential or wealth* or power*) adj1 (person* or people or figure* or leader or athlete* or player or bodybuilder or sport* or basketball or football or hockey or baseball or soccer or Olympian or singer* or songwriter* or musician* or band or group or rapper* or artist* or actor* or actress or star or Hollywood or Bollywood or Nollywood or dancer or writer or author or comedian or performer or model* or supermodel* or chef or philanthropist or politic* or president or minister or king or queen or prince* or monarch)))
14 3 and 6 and 9 and 12 and 13

Table S3: Rationale for meta-analysis inclusion and data processing

Boyland 2013<br>Intervention = Branded crisps commercial with celebrity endorser (Walker’s ad)<br>Comparison = Non-food commercial (control)<br>Excluded conditions =TV endorser in non-food context (Match of the Day) and savoury food commercial (not included)<br>Outcome = mean ad libitum consumption (kcal) of endorsed and non-endorsed snack, both were crisps (Walkers ready salted), but were labelled and verbally informed “Walker's” and “supermarket brand”, time ns.<br>Conversions= Converted from g to kcal g using published Walkers nutritional info. https://www.walkers.co.uk/crisps-range/walkers-crisps/ready-salted
Coates 2019<br>Intervention = Influencer food marketing with disclosure + influencer food marketing with no disclosure<br>Comparison = Influencer non-food marketing with no disclosure (control)<br>Outcome = mean ad libitum intake (kcal) of endorsed and non-endorsed snack, both were chocolate biscuits (McVitie’s digestives), but were labelled and verbally informed “McVitie's” and “Tesco's”, 5 minutes.
Coates 2019<br>Intervention = Influencer unhealthy food marketing (chocolate cookies)<br>Comparison = Influencer non-food marketing (control)<br>Excluded condition = Influencer healthy food marketing (banana)<br>Intake = mean ad libitum intake (kcal) of unhealthy snacks (jelly candy and chocolate buttons), 10 minutes.<br>Excluded outcome = mean ad libitum intake of healthy snacks (carrot batons and seedless white grapes).

Figure S1: Trim and fill analysis

```
. metatrim _ES _seES, egger

Note: default data input format (theta, se_theta) assumed.

Meta-analysis

       |  Pooled      95% CI         Asymptotic      No. of
Method |     Est    Lower    Upper  z_value  p_value   studies
-------+----------------------------------------------------
Fixed  |  45.720  25.293  66.146    4.387    0.000      3
Random |  56.351   8.500 104.201    2.308    0.021

Test for heterogeneity: Q=  9.762 on 2 degrees of freedom (p= 0.008)
Moment-based estimate of between studies variance = 1396.957

Trimming estimator: Linear
Meta-analysis type: Fixed-effects model

iteration |  estimate    Tn    # to trim     diff
----------+--------------------------------------
    1     |   45.720      4         1           6
    2     |   37.252      5         2           2
    3     |   14.405      5         2           1
    4     |   14.405      5         2           0

Filled
Meta-analysis

       |  Pooled      95% CI         Asymptotic      No. of
Method |     Est    Lower    Upper  z_value  p_value   studies
-------+----------------------------------------------------
Fixed  |  14.405  -2.319  31.129    1.688    0.091      5
Random |  14.405 -38.632  67.442    0.532    0.594

Test for heterogeneity: Q= 37.581 on 4 degrees of freedom (p= 0.000)
Moment-based estimate of between studies variance = 3218.284
```

Figure S2: **Bias assessment for experimental studies**

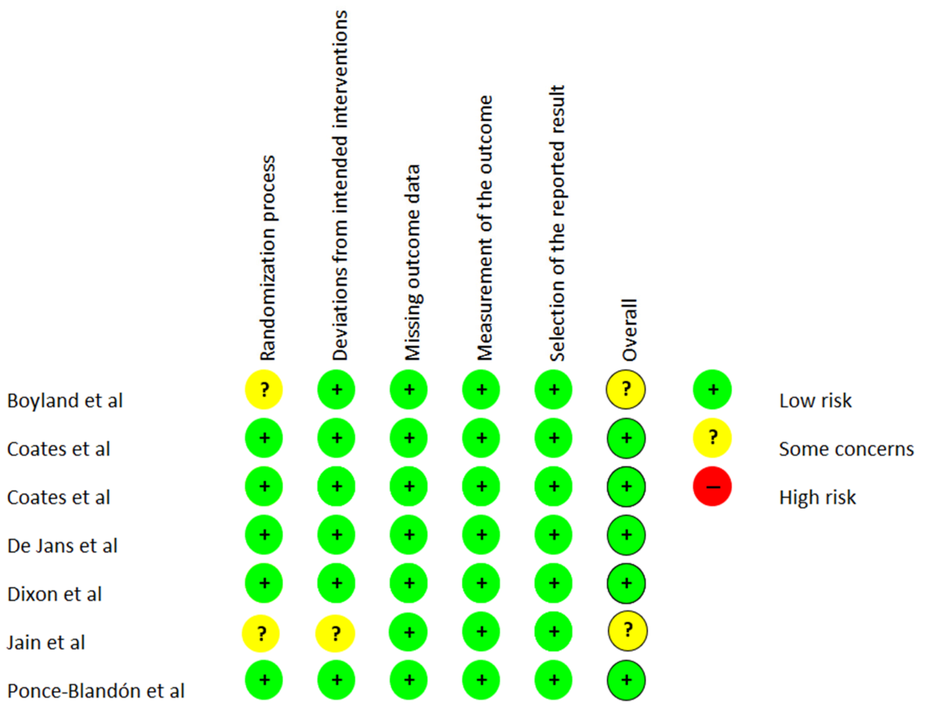

Supplement: Supplementary file 1 [file nutrients-14-00434-s001.zip › nutrients-1544815-supplementary.pdf]
